# Survey of water supply and assessment of groundwater quality in the suburban communes of Selembao and Kimbanseke, Kinshasa in Democratic Republic of the Congo

**DOI:** 10.1007/s40899-021-00592-y

**Published:** 2021-11-10

**Authors:** Michel L. Kapembo, Florent B. Mukeba, Periyasamy Sivalingam, Johnny B. Mukoko, Mathieu K. Bokolo, Crispin K. Mulaji, Pius T. Mpiana, John W. Poté

**Affiliations:** 1 Department of Chemistry, Faculty of Science, University of Kinshasa, B.P. 190, Kinshasa XI, Democratic Republic of the Congo; 2 Human Science Research Center (CRESH), 33, Avenue Comité Urbain, Commune de la Gombe, B.P 3474, Kinshasa/Gombe, Democratic Republic of the Congo; 3 Postgraduate and Research Department of Microbiology, Jamal Mohamed College, Tiruchirappalli, Tamil Nadu 620020 India; 4 Department of Biology, Faculty of Science, National Pedagogical University, Kinshasa, Democratic Republic of the Congo; 5 Faculty of Sciences, Institute F. A. Forel and Institute of Earth and Environmental Sciences, University of Geneva, Bd Carl-Vogt 66, 1211 Geneva 4, Switzerland

**Keywords:** Groundwater, Drinking water, Microbiological pollution, Epidemiology, Waterborne diseases, Human risk

## Abstract

In many suburban municipalities of developing countries, the household drinking water comes mainly from groundwater including, wells, streams and springs. These sources are vulnerable because poor hygienic conditions and sanitation prevail causing persistence and recurrent waterborne diseases. In this research, a survey study on water resource use and an epidemiological survey of waterborne diseases were conducted among users of water points and medical institutions in suburban communes of Selembao and Kimbanseke (Kinshasa, the Democratic Republic of the Congo). In addition, physicochemical (temperature, pH, O_2_, electrical conductivity, and soluble ions: Na^+^, K^+^, PO_4_^3−^, SO_4_^2−^, NO_3_^−^, NO_2_^−^) and bacteriological (FIB: faecal indicator bacteria) analyses of water from 21 wells and springs were performed according to the seasonal variations. FIB included *Escherichia coli* (*E. coli*), *Enterococcus* and Total Coliforms. The survey results indicate that more than 75% of the patients admitted to local medical institutions between 2016 and 2019 are affected by waterborne diseases, including typhoid fever, amoebic dysentery, diarrhoea, gastroenteritis disorders and cholera. Except for NO_3_^−^ in some sites, the water physicochemical parameter values are within WHO permissible limits for drinking/domestic water quality. On the contrary, the results revealed high FIB levels in water from unmanaged wells and springs during rainy and dry seasons. The microbiological pollution was significantly higher in the rainy season compared to the dry season. Interestingly, no FIB contamination was observed in water samples from managed/developed wells. The results from this study will guide local government decisions on improving water quality to prevent recurrent waterborne diseases.

## Introduction

The contaminated water sources used for the human purpose and poor sanitation are associated with transmission of diseases such as diarrhoea, cholera, hepatitis A, typhoid, dysentery and polio (Montgomery and Elimelech 2007; WHO 2011). Other diseases caused by contaminated water are transmissible to vulnerable communities (poor peoples) because they live in an environment accessible to breeding insect vectors that carry parasites such as paludism, filaria and trypanosomes (WHO 2017). The poor people are disadvantaged in that many of them live in water-deficient countries, mostly in sub-Saharan Africa, Asia and the Middle East. About 30% of the world’s population still do not have access to safe drinking water and 60% do not have reliable sanitation (WHO 2018). Globally, at least 2 billion persons use a drinking water source contaminated with faeces (WHO 2019). Every year, about 4 billion cases of diarrhoeal diseases are attributed to inadequate water, sanitation and hygiene and around 1 million of people are estimated to die each year from diarrhoea, most of them among children under 5 years of age in developing countries (WHO 2011, 2019). In developing countries, mainly in sub-Saharan African countries, Latina America and south of Asia, about 435 million persons drink water from unprotected wells and springs, and 144 million persons collect untreated surface water from lakes, ponds, rivers and streams (WHO 2017, 2019). According to Dey et al. (2017), the access to pit latrines in developing countries, the water sanitation and hygiene (WASH) situation has dramatically improved. However, the microbiological contamination from leakage in pit latrines cannot be excluded. A recent report by Bivins et al. (2021) showed that intermittent water supply (IWS) in a major city of India is associated with poor water quality and cause waterborne diseases. It has long been a major challenge for safe drinking water in many African countries (Adesakin et al. 2020). According to Ercumen et al. (2015), poor sanitation and FIB contamination in tube well waters are major causes of waterborne diseases in Mymensingh district, Bangladesh. In the Democratic Republic of the Congo (DRC), with an estimated population of 65.7 million inhabitants, despite the potential of its rich freshwater network, more than 75% of the people have no access to safe water (UNEP 2011). Polluted streams, groundwater/shallow wells and springs contaminated by micropollutants and pathogenic organisms are the most common domestic and drinking water sources for suburban and rural people (Kapembo et al. 2016, 2019; Nienie et al. 2017; Abanyie et al. 2020). Several causes can justify the pollution of these water resources, including the vulnerability of water points (lack of well water tightness), unsanitary conditions, contamination from septic tanks and latrines, the presence of uncontrolled landfills, wastewater runoff and open defecation (Banks et al. 2002; Longo 2009; Kapembo et al. 2016, 2019; Graham and Polizotto 2013; Abioye and Perera 2019; Owamah 2020). For this reason, it is necessary to examine the pollution status of the water used for human consumption.

Kinshasa is the largest and capital city of the DRC with more than 16 million inhabitants. About 75% of the population lives in suburban municipalities. The majority (more than 70%) has no access to safe water provided by the national society of water supply (Regideso). Household drinking water comes mainly from groundwater (including wells and springs) as well as urban streams. Sanitation and hygienic conditions in these municipalities are very worrying. Consequently, the suburban municipalities of Kinshasa are notorious for their recurrent outbreaks of disease, mainly waterborne diseases including, gastrointestinal, typhoid, cholera and other diarrheal diseases (UNEP 2011; EIES 2012; Kapembo et al. 2019). Our previous studies on the epidemiological survey and laboratory analysis of faecal indicator bacteria (FIB) in drinking water sources have been conducted in two suburban municipalities of Kinshasa, including Bumbu and Mont Ngafula (Kapembo et al. 2016, 2019). These studies indicated that waterborne diseases affected more than 60% of the patients admitted to local hospitals between 2013 and 2017. In addition, water sources in the previously studied wells are highly contaminated by FIB, including *Escherichia coli* (*E. coli*), *Enterococcus* (ENT) and Total Coliforms. These studies recommend further researches in other suburban municipalities of Kinshasa considering different criteria, including the density of population, personal hygiene, category of water sources, the frequency and number of users, waterborne disease epidemiology and economic situation of local people. Consequently, in this study, two suburban municipalities were selected: the municipalities of Selembao (335,581 inhabitants) and Kimbanseke (the most populous commune of Kinshasa with about 2 million inhabitants). The persons living in these municipalities are among the poorest inhabitants in Kinshasa city (EIES 2012). The neighbourhoods of these municipalities are well known for the lack of drinking water, sanitation services and electricity, and persistent and recurrent epidemics of waterborne diseases.

To our best knowledge, the data on the population waterborne diseases and the quality of water used for domestic purposes from these municipalities are still scarce. This research aims (1) to investigate water sources supply and associated waterborne diseases, and (2) to assess the seasonal variations of the water quality from wells and springs used by populations of these two municipalities for domestic purposes. The water quality assessment is based on determining water physicochemical characterisation including pH, electrical conductivity, dissolved oxygen, soluble ions (Na^+^, K^+^, PO_4_^3−^, SO_4_^2−^, NO_3_^−^, and NO_2_^−^) and the quantification of FIB including *Escherichia coli*, *Enterococcus*, and Total Coliforms. According to the World Health Organization guidelines for Drinking-water Quality (WHO 2017), the physicochemical parameters were selected. Contaminated water for domestic purpose is associated with high human risks (Owamah 2020; Burri et al. 2019; Hasan et al. 2019; Kayembe et al. 2018; Kapembo et al. 2016). Therefore, the epidemiological survey and water quality assessment are very important for preventing and reducing the long-term impact of water-related diseases in developing countries (WHO 2019).

## Materials and methods

### Study sites’ description

**Fig. 1:**
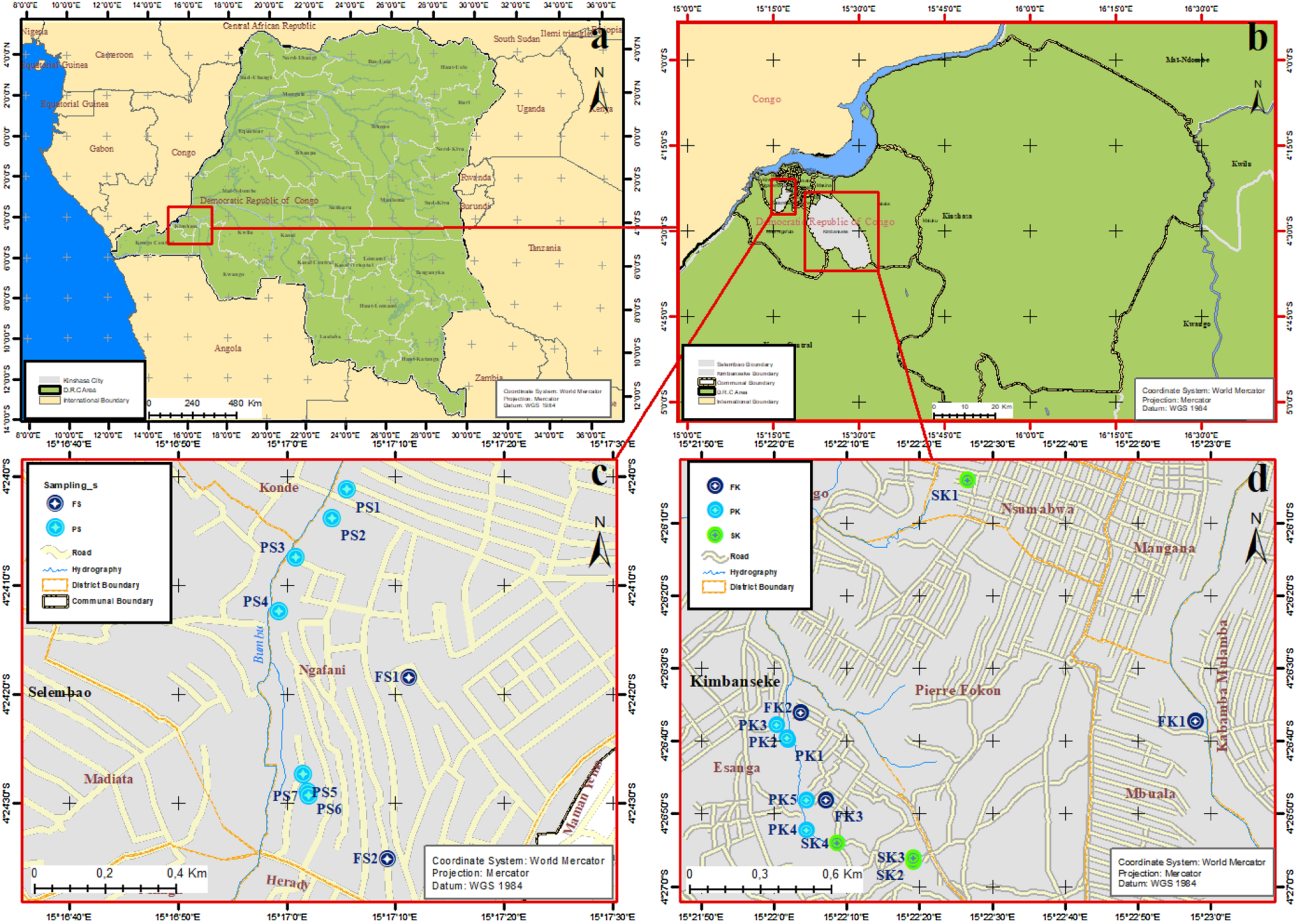
Adapted from Google maps of sampling site. **a** Democratic Republic of the Congo, **b** map showing the location of Kinshasa City in Democratic Republic of the Congo, **c** Selembao commune, districts of Ngafani (Sampling sites: FS1, FS2, PS1, PS2, PS3, PS4, PS5, PS6, and PS7) and **d** Kimbanseke commune, districts of Esanga (Sampling sites: FK1, FK2, FK3, PK1, PK2, PK3, PK4, PK5, SK1, SK2, SK3 and SK4)

This research was conducted in two suburban municipalities of the city of Kinshasa (Fig. 1); the municipalities of Selembao (Ngafani district) and Kimbanseke (Esanga district). These municipalities are characterised by rapid demographic growth and unplanned urbanisation. Many neighbourhoods of these municipalities are subject to frequent flooding and landslides, while hygiene and sanitation conditions are poor. Uncontrolled landfills are widespread and can also be used as open defecation sites. There no industrial activities in these areas, but the population practices intensive urban agriculture and animal husbandry.

The investigated districts (Ngafani and Esanga) are not connected to a public water supply network (Regideso), and there is a lack of safely managed sanitation services. Wells and springs constitute the primary sources of water for domestic purposes; there are several managed (developed/protected) and unmanaged (unprotected) wells and springs that are the primary domestic (drinking, cooking, and washing) sources of water supply. These districts were selected according to the results and recommendations from our previous studies (Kapembo et al. 2016, 2019).

### Survey study on water management and prevalence of waterborne diseases

The survey study for the management/use of water from wells and springs and the prevalence of waterborne diseases in studied municipalities were performed between 2016 and 2019 as described by Kapembo et al. (2019). The survey comprised field observations, interviews with the local population and medical institutions and water sources managers. For the management/use of water resources, 200 households per district were selected to obtain information concerning the water supply mode by users, latrines location, wells and springs management, socio-demographic systems, and sanitation conditions of users. All interviewed persons (children and adults) supply their water from studied districts. The selection of survey participants was performed according to the age and sex of users (mainly children under 15 years old and women) in the sites studied. The survey study was conducted among two age groups: 59% under 15 years old (with the age range of 9–15 years and an average of 13 years old) and 41% over 15 years of age (with age range of 16–35 years and an average of 23 years old). Among the surveyed population, 60% were females and 40% were males.

For the prevalence of waterborne diseases (including typhoid fever, amoebic dysentery, filariasis, diarrhoea, gastroenteritis disorders and cholera) in households from studied municipalities, the survey was carried out in the form of a questionnaire to the appropriate local medical institutions (4 from Ngafani district (Selembao) and 5 from Esanga district (Kimbanseke).

### Water sampling procedure

**Fig. 2:**
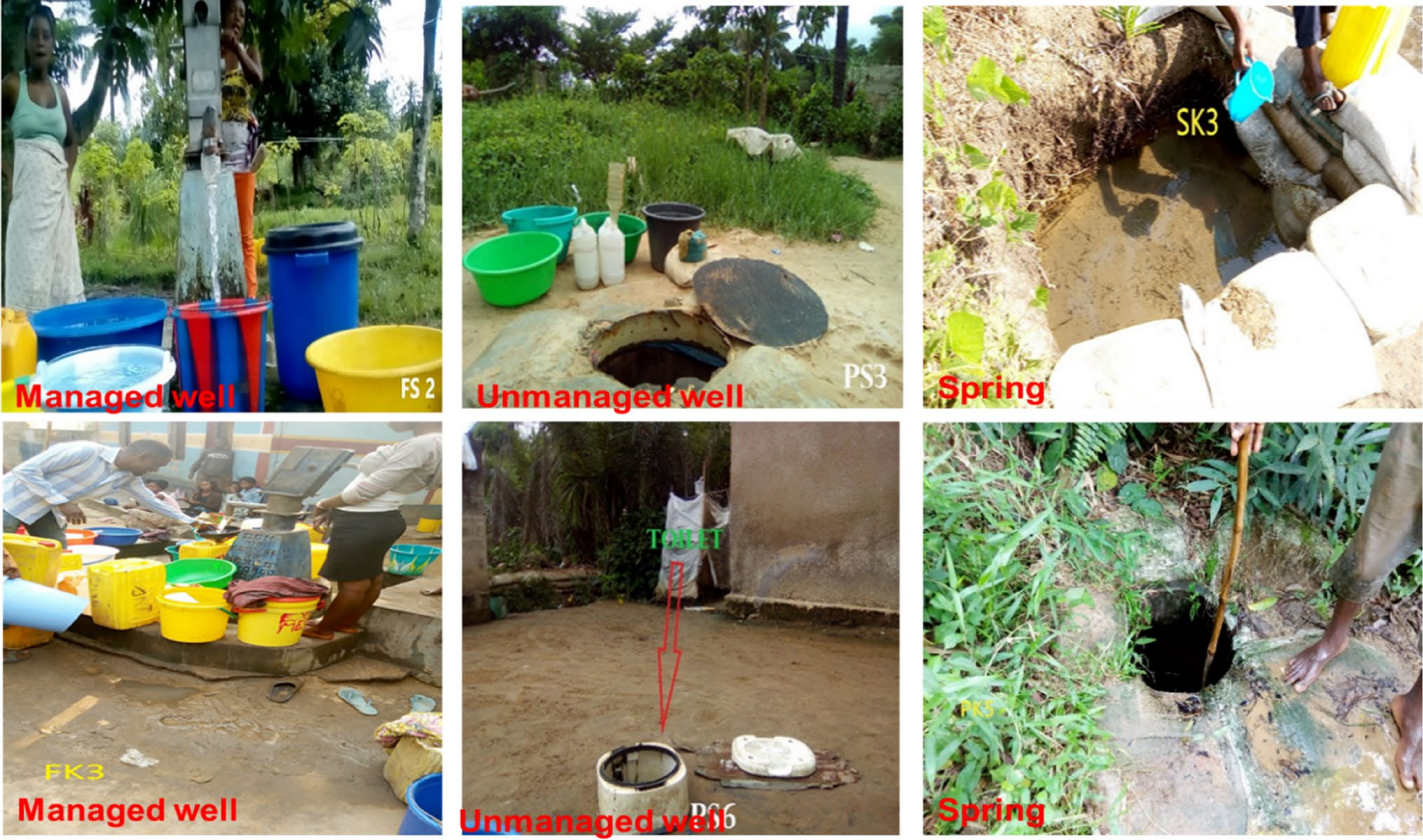
Some sampling points (FS2, PS6, PS3) of the municipality of Selembao and (PK6, SK3 and FK3) for Kimbanseke (photos taken by M. Kapembo and F. Mukeba in October 2019)

Water sampling from wells and springs took place during the dry season (May–August, 2018) and (June–September 2019) in Selembao (Ngafani district) and Kimbanseke (Esanga district), respectively, and during the rainy season (January–April 2019) and (October–December 2019) from Selembao and Kimbanseke municipalities, respectively. The samples are labelled as follows (Fig. 1): (1) Selembao township: FS1–FS2 (managed/developed wells); PS1–PS7 (unmanaged wells), and (2) Kimbanseke municipality: FK1–FK3 (managed/developed wells); PK1–PK5 (unmanaged wells); SK1–SK4 (springs) (Fig. 2). The GPS location of sampling sites, the depth of the water level in the wells, and the number of users are reported in Table 1.

**Table 1:** Well and stream GPS locations, depth, and number of users

Sampling site	Longitude	Latitude	Well depth (m)		Colour	No users	Construction year
			Dry season	Rainy season			
FS1	15° 17′ 11.2″	4° 24′ 18.5″	22	–	Clear	± 1200	2019
FS2	15° 17′ 09.3″	4° 24′ 35.2″	35	–	Clear	± 2500	2018
FK1	15° 22′ 58.0″	4° 26′ 37.3″	40	–	Clear	± 2500	2015
FK2	15° 22′ 03.7″	4° 26′ 36.1″	28	–	Clear	± 1500	2016
FK3	15° 22′ 07.1″	4° 26′ 48.1″	30	–	Clear	± 1000	2014
PS1	15° 17′ 05.5″	4° 24′ 1.2″	3	1	Soft	± 200	2016
PS2	15° 17′ 04.1″	4° 24′ 03.8″	3	1.5	Clear	± 300	2017
PS3	15° 17′ 00.8″	4° 24′ 07.4″	5	3	Clear	± 300	2016
PS4	15° 16′ 59.2″	4° 24′ 12.4″	3	2	Soft	± 100	2016
PS5	15° 17′ 01.5″	4° 24′ 27.4″	7	5	Clear	± 400	2017
PS6	15° 17′ 01.9″	4° 24′ 29.0″	3	2	Clear	± 200	2016
PS7	15° 17′ 02.0″	4° 24′ 29.4″	3	2	Clear	± 150	2017
PK1	15° 22′ 01.8″	4° 26′ 39.8″	5	5	Clear	± 50	2015
PK2	15° 22′ 01.7″	4° 26′ 39.6″	10	9	Soft	± 80	2018
PK3	15° 22′ 00.3″	4° 26′ 37.8″	4	4	Soft	± 100	2016
PK4	15° 22′ 04.4″	4° 26′ 52.2″	8	7	Clear	± 80	2013
PK5	15° 22′ 04.4″	4° 26′ 48.1″	6	4	Clear	± 200	2019
SK1	15° 22′ 26.6″	4° 26′ 04.1″	1	1	Clear	+ 500	2017
SK2	15° 22′ 19.1″	4° 26′ 56.6″	1	1	Clear	± 500	2017
SK3	15° 22′ 19.2″	4° 26′ 56.1″	0.5	1	Clear	± 300	2018
SK4	15° 22′ 08.7″	4° 26′ 54.1″	0.8	1	Soft	± 150	2018

FS1–FS2 (managed/developed wells) and PS1–PS7 (unmanaged wells) from the district of Ngafani (Selembao commune). FK1–FK3 (managed/developed wells), PK1–PK5 (unmanaged wells) and SK1–SK4 (springs) from the district of Esanga (Kimbanseke commune)

For the managed wells, water samples were collected directly from outlet pipes. As for unmanaged wells, water was collected by a craft device made of a 1 L clean polyethylene bottle attached to a rope (Kapembo et al. 2016). For springs, water was collected manually by directly dipping a polypropylene plastic container (500 mL). Once collected, samples were stored in an icebox and transported to the laboratory for analysis within 48 h.

### Physicochemical parameter analysis

The water physicochemical parameters including, temperature (T), pH, dissolved oxygen (O_2_) and electrical conductivity (EC), were measured in situ using a Multi 350i (WTW, Germany). The concentration of dissolved ions (Na^+^, K^+^, PO_4_^3−^, SO_4_^2−^, NO_3_^−^, and NO_2_^−^) was measured using an Ion Chromatography (Dionex ICS-3000, Canada) according to the method described by Mavakala et al. (2016). The certified water material (CRM, Ontario-99, Water Research Institute, Canada) was used to verify the instrument’s accuracy. The CRM results were within the acceptance range on the CRM certificate.

### Faecal indicator bacteria analysis in water samples

The faecal indicator bacteria (including *Escherichia coli* (*E. coli*), *Enterococcus* (ENT) and Total Coliforms (TC) were quantified in water samples according to the international standard methods for water quality determination using the membrane filtration method (APHA 2005). Briefly, for each sample, triplicates of 100 mL of water were passed through a 0.45 mm filter (Sartorius stedim, biotech, Germany), and then placed on different selective culture media (Biolife, Italiana), using the following incubation conditions: for *E. coli* bacteria analysis: each water sample was inoculated into Tryptone Soy Agar (TSA) medium and incubated at 37 °C for 4 h, and then transferred to Tryptone Bile X-Gluc Agar (TBX) medium at 44 °C for 24 h; for ENT bacteria analysis: each water sample was inoculated into Slanetz Bartley Agar (SBA) medium and incubated at 44 °C for 48 h, and then transferred into Bile Aesculin Agar (BAA) medium at 44 °C for 4 h, and into Endo agar medium, incubated at 35° C during 24 h for TC. The results are expressed as colony-forming units per 100 mL of water sample (CFU 100 mL^−1^). The reproducibility of the whole experimental procedure was tested by employing triplicates. Field and laboratory controls were performed as described in our previous studies (Nienie et al. 2017; Kapembo et al. 2016; Kilunga et al. 2016).

### Statistical analysis

All water samples analyses were carried out in triplicate for each set of conditions. In addition, three plates per dilution were performed for FIB quantification to establish plant count standard deviation (Kayembe et al. 2018; APHA et al. 2012). The statistical treatment of the data was realised using SigmaStat 11.0 (Systat Software, Inc.). The data were subjected to a Spearman’s Rank-order correlation test to investigate possible relationships using RStudio statistical software, Version 1.3.1093, © 2009–2020 RStudio, PBC.

## Results and discussion

### Survey of water source management

From the field observation and measurement, the distance between water points (wells and springs) and the dwelling place, in both municipalities varied considerably from 0 to 120 m. The distance between latrines and wells are, in most cases, ranging between 0 and 10 m. Developed wells and springs are the most of household water supply systems, but with limited numbers and costly. Wells constitute the preferable and access water sources for more than 80% of persons in studied municipalities. 41% of the population providing household water supply are children under 15 years old, and 59% were over 15 years old. 99% of the children who fetched water from wells did not wash their hands after defecation or drawing water. 100% of over 15 years old do not wash their hands before drawing water and defecation. 6% of under 15 years had already defecated near a water source, especially during rainy. 80% of children under 15 years of age surveyed say they evaluate the potability of water by its colour (colourless) and odour (odourless). 75% of children under 15 years of age admit having experienced diarrhoea or stomach aches after drinking this water, especially water from unmanaged wells and springs.

### Epidemiologic survey associated to waterborne diseases

The data obtained from the medical institutions of Selembao and Kimbanseke municipalities concerning the prevalence of the diseases for 2016–2019 are reported in Table 2. These data are not representative because the most people do not go to the medical institutions (except in severe cases) due to a lack of financial means. However, the data show a high prevalence of waterborne diseases in Ngafani (Selembao) and Esanga (Kimbanseke) districts. For example, Ngafani (Selembao) community is characterised by a high prevalence of typhoid fever, with 782 and 880 cases during 2018 and 2019, respectively. Esanga (Kimbanseke) district is characterised by a high prevalence of diarrhoea with 300, 279, 311 and 314 cases during 2016, 2017, 2018 and 2019, respectively. Other cases of associated water diseases are not negligible, e.g. 1117 and 325 cases of malaria were noticed in the Ngafani district during 2017 and 2018, respectively. While for gastroenteritis and typhoid fever, a substantial increase is observed in Ngafani district, in contrast, in Esanga district, there is an annual decrease in case numbers. The improvement of the water supply can probably explain this by an increase in the number of managed springs and wells [about 40% of springs and wells are developed (managed/protected)] or limited access to medical institutions in this district. The prevalence of waterborne diseases by age group for June/October 2018 for the Ngafani district is shown in Table 3. The results showed that the age group below 15 years is more affected by waterborne diseases. It can be noticed that about 59% of the Congolese population is in the 0–19 age group (MISC-RDC 2010).

**Table 2:** Prevalence of the diseases during the period of 2018–2019 in the districts of Ngafani (Selembao) (Data source: Medical formations from Ngafani in Selembao, taken by Michel Kapembo in March 2019) and Esanga (Kimbanseke) (Data source: Medical formations from Esanga in Kimbanseke, taken by Florent Mukeba in March 2019)

Diseases	2016		2017		2018		2019	
	Ngafani	Esanga	Ngafani	Esanga	Ngafani	Esanga	Ngafani	Esanga
Typhoid fever	38	6	ND	ND	782	4	880	8
Paludism	6	16	1117	8	325	30	147	68
Amoebic dysentery	2	9	20	28	20	13	9	26
Filariasis	4	3	ND	7	ND	13	ND	7
Gastroenteritis disorders	2	21	1	26	6	18	10	10
Cholera	1	1	0	1	0	6	2	3
Diarrhoea	98	300	269	279	86	311	64	314

*ND* data no obtained

**Table 3:** Average prevalence of waterborne diseases (according to age bracket) during the period of January–October 2018 in the districts of Ngafani (Data source: Medical formations from Selembao commune, taken by Michel Kapembo in February 2019)

Diseases	0–11 months	1–4 years	5–14 years	More than 15 years	Average
Paludism	35.11	39.79	41.03	52.66	42.15
Typhoid fever	1.77	2.33	6.55	8.85	4.88
Amebic dysentery	1.04	2.85	3.12	5.82	3.21
Gastroenteritis disorders	1.32	0.99	0.74	0.89	0.99
Filariasis	2.18	1.94	0.75	3.45	2.08
Diarrhoea	3.50	2.97	2.15	2.32	2.74

Several studies conducted in similar environments demonstrated that lack of access to drinking water is the leading cause of the emergence of waterborne diseases and impacts health by causing acute infectious diarrhoea and repeat or chronic diarrhoea episodes (e.g. Hunter et al. 2010; Kouam 2013). Prüss-Ustun and Carvalan (2006) demonstrated that the lack of water, sanitation, and hygiene system is responsible for 29% of diarrhoeal diseases in developing countries, mainly in cities with a growing population. Fitzwater et al. (2011) noted that about 88% of diarrhoeal diseases are attributed to poor water quality and hygiene. Our previous study (Kapembo et al. 2019) demonstrated 61% of people living in suburban municipalities of Mont Ngafula (Kinshasa) suffered from waterborne diseases: diarrhoea (11%); gastroenteritis disorders (7%); typhoid fever (5%); amoebic dysentery (5%); filariasis (4%) and cholera (less than 1%).

### Water physicochemical characteristics

The results of water physicochemical parameters including temperature (*T*), pH, electrical conductivity (EC) and dissolved oxygen (O_2_) according to the seasonal variations are reported in Table 4. The values of *T*, pH and EC observed in all sampling sites in dry and rainy seasons are generally within the recommended values set by World Health Organization Guidelines for Drinking-water Quality (WHO 2011, 2017). The water temperature was higher during the rainy season with the maximum values ranged between 26.3 and 28.8 °C and lower during the dry season with the values ranging from 23.9 to 26.9 °C. The pH ranged from 5.2 to 6.9 and 5.3–7.8 during the dry and rainy seasons, respectively. The EC varied considerably with sampling sites and seasonal variations. The maximum value of 704 µS cm^−1^ was observed in the site PK3 during the dry season and the minimum value of 24 µS cm^−1^ in PS5 during the rainy season. These values are comparable with other studies performed in similar environments under tropical conditions (Amanial 2015; Nienie et al. 2017) and lower than the values ranged between 605 and 1547 24 µS cm^−1^ observed by Kapembo et al. (2016) in wells from the municipalities of Bumbu (Kinshasa). The O_2_ values were higher in the developed wells FK1–FK3, ranging between 4.1 and 5.0 mg L^−1^ and 5.1–6.8 mg L^−1^ during the dry and rainy seasons, respectively. These values are within WHO Guidelines for Drinking-water Quality (4–7 mg L^−1^). Except for the site SK3 during the dry season with the value of 4.9 mg L^−1^, other sampling sites present the lowest values of O_2_ ranged between 1.1 and 3.6 mg L^−1^.

**Table 4:** Physicochemical parameters [temperature *T*, pH, Electrical conductivity (EC), and dissolved oxygen (O_2_)] of water samples from wells and springs during the dry (dry) and rainy (rainy) season

Sampling sites	*T* (°C)		pH		EC (μs cm^−1^)		O_2_ (mg L^−1^)	
	Dry	Rainy	Dry	Rainy	Dry	Rainy	Dry	Rainy
FS1	24.6	26.4	6.1	5.9	135	94	2.3	1.9
FS2	24.1	25.8	6.4	6.2	325	112	3.5	2.3
FK1	26.5	28.1	5.6	6.3	264	197	4.1	5.1
FK2	26.8	28.5	6.1	4.6	158	178	5.0	6.8
FK3	26.2	28.0	5.3	5.6	138	182	4.7	6.2
PS1	26.5	27.3	6.1	5.8	342	85	2.7	2.9
PS2	25.8	27.9	6.5	5.7	258	132	3.2	3.5
PS3	24.9	26.7	6.8	6.2	105	74	1.5	2.3
PS4	26.1	26.3	6.9	5.7	470	123	3.0	2.7
PS5	25.3	27.5	5.9	6.4	85	24	1.1	2.2
PS6	24.8	27.7	6.5	5.8	189	135	3.4	2.9
PS7	26.3	26.6	6.7	5.9	435	289	2.9	3.1
PK1	23.9	27.3	6.1	7.4	563	82	3.1	2.4
PK2	26.6	27.6	5.9	7.8	206	85	2.2	3.0
PK3	26.5	27.4	6.3	6.7	704	98	2.5	3.3
PK4	25.2	28.0	6.6	5.3	48	88	1.8	2.9
PK5	24.6	28.1	5.6	5.6	304	66	3.6	3.2
SK1	26.4	27.8	5.7	6.6	115	74	1.9	1.4
SK2	26.9	27.2	5.2	6.7	363	82	3.7	3.1
SK3	26.5	27.1	5.5	7.2	458	72	4.9	3.0
SK4	24.3	28.8	5.3	7.0	62	78	2.5	3.2
WHO regulation^a^	12–25		6.5–9.5		200–800		4–6	

FS1–FS2 (managed/developed wells) and PS1–PS7 (unmanaged wells) from the district of Ngafani (Selembao commune)

FK1–FK3 (managed/developed wells), PK1–PK5 (unmanaged wells) and SK1–SK4 (springs) from the district of Esanga (Kimbanseke commune)

^a^Limit recommended by World Health Organization Guidelines for Drinking-water Quality (WHO 2017)

The concentration of soluble ions (Na^+^, K^+^, PO_4_^3−^, SO_4_^2−^, NO_3_^−^, and NO_2_^−^) in water samples are reported in Table 5. Except for NO_3_^−^ in 7 sites (PS1, PS4, PK1, PK2, PK4, SK2, SK4) during the rainy season, the concentration of other ions in water samples from all studied sites meets the WHO guidelines for Drinking-water Quality (WHO 2017) during both dry and rainy seasons. The managed/developed wells present the lowest concentration with values ranging between 1.3–6.3 mg L^−1^ and 8.5–12.4 6 mg L^−1^ during dry and rainy seasons. In unmanaged/undeveloped wells, the concentration of NO_3_^−^ varied significantly according to the sampling sites (*P* ˂ 0.05), with the values ranged between 1.4–43.6 and 9.8–83.1 mg L^−1^ during the dry and rainy seasons, respectively. The same tendency was observed in springs, with the values ranged between 2.3–47.8 mg L^−1^ and 7.5–68.2 mg L^−1^ during the dry and rainy seasons, respectively.

**Table 5:** Concentration of soluble ions in water samples from wells and springs during the dry (dry) and rainy (rainy) season

Sampling sites	Na^+^ (mg L^−1^)		K^+^ (mg L^−1^)		PO_4_^3−^(mg L^−1^)		SO_4_^2−^ (mg L^−1^)		NO_3_^−^(mg L^−1^)		NO_2_^−^(mg L^−1^)	
	Dry	Rainy	Dry	Rainy	Dry	Rainy	Dry	Rainy	Dry	Rainy	Dry	Rainy
FS1	92.61	36.42	5.46	2.37	0.09	0.01	103.24	78.66	6.33	8.52	0.01	0.01
FS2	78.56	58.90	4.88	2.19	0.83	0.07	94.32	19.45	1.32	12.40	0.01	0.01
FK1	98.34	60.11	7.53	2.34	0.71	0.35	132.12	77.66	7.32	10.52	0.01	0.01
FK2	103.20	51.14	8.52	3.17	0.88	0.62	215.32	141.27	5.41	9.06	0.01	0.01
FK3	99.6	52.8	8.59	6.4	0.56	0.37	145.89	85.9	6.25	11.3	0.02	0.02
PS1	28.64	17.44	8.50	6.11	0.01	0.02	128.15	31.01	25.48	72.17	0.02	0.09
PS2	19.77	11.50	4.23	2.89	0.12	0.03	24.75	12.43	9.55	16.24	0.01	0.04
PS3	31.01	24.35	2.89	3.56	1.04	0.07	9.07	6.06	9.98	10.12	0.02	0.05
PS4	27.22	12.84	6.11	7.55	0.94	0.02	12.02	4.75	21.52	65.80	0.05	0.15
PS5	44.10	31.50	4.99	2.93	0.45	0.02	7.88	6.55	12.56	24.58	0.01	0.01
PS6	9.96	10.11	7.37	3.19	0.78	0.04	11.23	7.88	28.01	31.04	0.06	0.09
PS7	52.44	42.31	10.47	7.51	1.03	0.02	20.05	12.92	1.38	9.78	0.01	0.05
PK1	39.86	23.44	2.92	0.66	0.38	0.01	77.77	23.44	38.09	74.14	0.03	0.99
PK2	7.91	2.14	3.64	1.04	0.54	0.33	139.44	101.03	43.61	83.11	0.77	1.02
PK3	78.81	69.56	3.14	1.19	0.95	0.99	134.11	76.15	29.16	35.09	0.01	0.03
PK4	10.22	8.77	2.06	0.99	0.09	0.01	111.09	45.22	32.53	75.12	0.02	0.07
PK5	32.73	21.17	3.71	1.24	0.71	0.03	13.65	8.91	7.90	28.41	0.02	0.07
SK1	53.41	32.11	8.32	4.22	0.32	0.21	49.33	22.45	2.30	7.45	0.01	0.01
SK2	18.76	6.58	2.34	0.39	0.98	0.99	200.03	64.49	47.84	68.24	0.01	0.87
SK3	27.12	3.33	6.45	2.22	0.56	0.12	99.05	45.08	4.58	10.17	0.01	0.04
SK4	22.53	9.99	3.56	1.18	1.15	0.55	88.26	42.14	31.15	59.44	0.01	0.96
WHO regulation^a^	10–100		15		≤ 0.5		500		50		3	

FS1–FS2 (managed/developed wells) and PS1–PS7 (unmanaged wells) from the district of Ngafani (Selembao commune). FK1–FK3 (managed/developed wells), PK1–PK5 (unmanaged wells) and SK1–SK4 (springs) from the district of Esanga (Kimbanseke commune)

^a^Limit recommended by World Health Organization Guidelines for Drinking-water Quality (WHO 2017)

Several recent studies have discussed the nitrate level in groundwater and its potential human health risks (Li et al. 2021; Adimalla and Qiana 2021). Nitrate and its compounds have occurred naturally in the aquatic environment. However, the high concentration of NO_3_^−^ observed in studied groundwater can be explained by several aspects, including infiltration of water from urban agriculture using fertilisers, the permeability of the unsaturated zone, aquifer depth, leaking septic tanks and unimproved sanitation systems, as well as excrement from livestock and uncontrolled landfills near the wells and streams (Banks et al. 2002; Balbus and Embrey 2002; Abdelaziz et al. 2007; Sacchi et al. 2013; Kapembo et al. 2016).

### Microbiological quality of water from wells and springs

The microbiological quality of water samples collected from wells and springs during both the rainy and dry seasons are presented in Table 5. The FIB (*E. coli*, ENT and FC) levels in water samples varied significantly according to sampling sites and seasonal variations (*P* ˂ 0.05). The water pollution was significantly higher in the rainy season compared to the dry season. In the managed/developed wells during the dry season, the average values (expressed in CFU 100 mL^−1^) ranged from 0 to 32, 0–48 and 0–19 were observed for *E. coli*, ENT and TC, respectively (Table 6). During the rainy season, the average values (expressed in CFU 100 mL^−1^) ranged from 0 to 90, 0–89 and 0–38 were observed for *E. coli*, ENT and TC, respectively. Surprisingly, no faecal contamination (presence of *E. coli*, ENT and TC) was observed in water samples collected from 3 of 5 managed/developed wells during dry and rainy seasons. These results suggest the total absence of faecal contamination in the wells (FS1, FK1 and FK3), indicating that the water from these wells can be used for domestic purposes according to WHO drinking water regulations regarding microbiological quality (Eu 2020; WHO 2020). These wells were built by NGOs and managed by individuals (considered private wells), requiring a water supply tax. This constitutes a great challenge for very low-income users.

**Table 6:** Average *Escherichia coli*, *Enterococcus*, and Total Coliform quantification in wells and stream during the dry season (dry) and rainy season (rainy)

Sampling sites	*E. coli* (CFU ± SD × 10^2^ 100 mL^−1^)		ENT (CFU ± SD × 10^2^ 100 mL^−1^)		TC (CFU ± SD × 10^3^ 100 mL^−1^)	
	Dry	Rainy	Dry	Rainy	Dry	Rainy
Managed wells						
FS1	0	0	0	0	0	0
FS2	0.32 ± 0.03	0.90 ± 0.35	0.48 ± 0.09	0.89 ± 0.12	0.19 ± 0.04	0.38 ± 0.11
FK1	0	0	0	0	0	0
FK2	0	0.03 ± 0.00	0	0.05 ± 0.00	0	0.09 ± 0.02
FK3	0	0	0	0	0	0
Unmanaged wells						
PS1	2.21 ± 0.18	20.01 ± 1.12	9.08 ± 1.31	28.01 ± 2.21	11.04 ± 1.04	35.30 ± 3.51
PS2	0.03 ± 0.02	0.21 ± 0.06	0.11 ± 0.09	0.98 ± 0.15	0.09 ± 0.01	0.64 ± 0.05
PS3	0.41 ± 0.07	0.81 ± 0.15	0.12 ± 0.03	1.12 ± 0.06	0.17 ± 0.02	0.31 ± 0.05
PS4	11.21 ± 0.05	19.31 ± 1.22	22.10 ± 0.51	16.11 ± 1.22	35.12 ± 3.97	79.12 ± 3.77
PS5	0.09 ± 0.01	0.03 ± 0.00	0.12 ± 0.08	0.19 ± 0.03	0.17 ± 0.06	0.29 ± 0.07
PS6	7.03 ± 0.39	29.07 ± 8.25	8.31 ± 2.51	10.52 ± 1.75	2.83 ± 0.32	14.32 ± 2.13
PS7	3.11 ± 0.12	24.06 ± 3.18	6.03 ± 1.15	28.13 ± 2.59	7.06 ± 1.48	13.42 ± 6.04
PK1	2.43 ± 0.35	12.08 ± 0.77	3.13 ± 0.77	15.18 ± 7.16	2.03 ± 0.04	17.22 ± 6.12
PK2	7.33 ± 1.11	29.36 ± 2.14	6.99 ± 2.01	16.10 ± 2.44	38.04 ± 2.17	79.03 ± 8.33
PK3	2.63 ± 0.44	29.36 ± 2.14	1.98 ± 0.22	32.08 ± 1.11	3.20 ± 0.18	19.03 ± 3.33
PK4	3.55 ± 0.99	22.44 ± 3.11	3.96 ± 1.02	43.10 ± 2.22	7.18 ± 0.92	14.99 ± 4.15
PK5	1.42 ± 0.13	45.12 ± 3.22	1.12 ± 0.25	56.29 ± 4.13	1.32 ± 0.81	24.44 ± 3.11
Streams						
SK1	0	0.03 ± 0.00	0	0.08 ± 0.01	0	0.07 ± 0.02
SK2	2.55 ± 0.33	4.37 ± 1.08	2.69 ± 0.47	8.15 ± 1.09	9.06 ± 1.75	10.03 ± 3.37
SK3	0.06 ± 0.01	0.90 ± 0.13	0.04 ± 0.00	0.78 ± 0.17	0.08 ± 0.01	0.24 ± 0.01
SK4	4.32 ± 0.76	6.50 ± 0.92	3.35 ± 0.18	9.88 ± 1.03	1.13 ± 0.04	7.11 ± 2.13
EU/WHO^a^	0		0		0	

*E. coli*: *Escherichia coli*; *ENT*: *Enterococcus*; *TC* Total Coliform; ± SD: standard deviation

^a^EU and World Health Organization Guidelines for Drinking-water Quality 0 CFU 100 mL^−1^, for *E. coli*, ENT and TC (EU 2020; WHO 2020)

For unmanaged/undeveloped wells and springs, the microbiological quality of water samples was inferior in both dry and rainy seasons and varied significantly according to sampling sites and seasonal variations (*P* ˂ 0.05). The FIB average values during the dry season ranged from (0.03–11) × 10^2^, (0.11–22) × 10^2^, and (0.09–38) × 10^2^ CFU 100 mL^−1^ for *E. coli*, ENT, and TC, respectively. During the rainy season, the average values ranged from (0.03–45) × 10^2^, (0.19–56) × 10^2^, and (0.29–79) × 10^2^ CFU 100 mL^−1^ for *E. coli*, ENT, and TC, respectively. These results indicate that water samples from all studied wells and springs are heavily polluted with FIB and did not meet the WHO guidelines for domestic use water, which recommends 0 CFU 100 mL^−1^ for *E. coli*, ENT and TC (Eu 2020; WHO 2020). Similar results were observed in a previous study conducted in Guinea-Bissau in shallow wells’ water samples, West Africa, reported that diarrhoea attributed 11.5% of all medical cases and of which most were children aged < 15 during the onset of the wet season (Bordalo and Savva-Bordalo 2007). The microbiological quality of water from springs was also poor, mainly during the rainy season. The FIB average values ranged from 0 to 432, 0–335 and 0–113 CFU 100 mL^−1^ for *E. coli*, ENT and TC, respectively. During the rainy season, the average values (expressed in CFU 100 mL^−1^) ranged from 3 to 650, 8–988 and 7–1000 for *E. coli*, ENT and TC, respectively. The high bacterial load observed in spring water during rainy season was probably a result of agricultural activities by the community, open defecation and runoffs from farmland into springs (Adesakin et al. 2020). Interestingly, no presence of FIB (*E. coli*, ENT, TC) in the water samples from the spring SK1 collected during the dry season was observed, suggesting the total absence of water faecal contamination in compliance with drinking water regulations (Eu 2020; WHO 2020). Indeed, a previous study performed by the other authors in a similar environment (Kikwit, DRC) indicated that FIB does not contaminate some wells and streams during the dry season (Nienie et al. 2017).

This study demonstrated that microbiological analysis of water samples from 100% of unmanaged/undeveloped wells and springs (except for spring KS1 during the dry season) are highly contaminated with faecal material. Consequently, the water from these sources is likely to contain pathogenic organisms responsible for water-related diseases such as gastrointestinal illnesses, typhoid, cholera, and other diarrhoeal diseases (EU 2020; WHO 2011; US EPA 2000; Haile et al. 1999; Noble et al. 2004; Davis et al. 2005). The deterioration of the microbiological water quality (mainly during the rainy season) can be explained by several causes, including the absence of toilet facilities (including open defecation and distance between toilet and water sources), percolation of contaminated surface soils during rain events, infiltration from toilet located near wells and springs, and direct contamination by users (Kayembe et al. 2018; Kapembo et al. 2016, 2019). Similar results were also observed in the researches performed in developing countries (sub-Sahara, Latina America and south of Asia) under tropical conditions (e.g. Owamah 2020; Burri et al. 2019; Hasan et al. 2019).

### Statistical correlation

Spearman’s rank-order correlation was carried out to identify a possible relationship between analysed parameters. The results are presented in Tables 7 and 8 for rainy and dry seasons, respectively. In general, no significant correlation was observed between physicochemical parameters and bacteriological (*E. coli*, ENT, TC) parameters during both the rainy and dry seasons. These results suggest that analysed physicochemical and bacteriological parameters of water can originate from different sources (Poté et al. 2009; Haller et al. 2009). However, a strong mutually positive correlation was observed between *E. coli*, ENT and TC during both the rainy and dry seasons; e.g. during the rainy season (Table 7): *E. coli* and ENT (*R* = 0.88, *P* < 0.001), and during dry season (Table 8): *E. coli*, ENT and TC (0.82 < *R* < 0.90, *P* < 0.05), ENT and TC (*R* = 0.79, *P* > 0.05). These results indicate that *E. coli*, ENT and TC could originate from common sources, influence bacterial growth and are carried into wells by common transporters (Haller et al. 2009; Poté et al. 2009; Kilunga et al. 2016; Adesakin et al. 2020). The same tendency was observed in our previous studies performed in a similar environment (Nienie et al. 2017; Kapembo et al. 2016, 2019).

**Table 7:** Spearman’s rank-order correlation of selected parameters [parameters include physicochemical parameters (pH, temperature (*T* °C), electrical conductivity (EC), dissolved oxygen (O_2_) and soluble ions (Na^+^, K^+^, PO_4_^3+^, SO_4_^2−^, NO_3_^−^ and NO_2_^**−**^) and faecal indicator bacteria (FIB): *Escherichia coli* (*E. coli*), *Enterococcus* (ENT), and Total Coliforms (TC). Significant coefficients (*P* < 0.05) are in bold] in water from wells analysed in rainy season

	*T* (°C)	pH	EC	O_2_	Na^+^	K^+^	PO_4_^3+^	SO_4_^2−^	NO_3_^−^	NO_2_^−^	*E. coli*	ENT	TC
*T* (°C)		− 0.102	− 0.020	0.518	− 0.130	− 0.620	− 0.090	− 0.186	0.103	0.175	0.077	0.125	− 0.166
pH			− 0.399	− 0.427	− 0.332	− 0.321	0.332	0.270	0.322	0.670	− 0.007	− 0.139	0.206
EC				0.555	0.388	0.388	− 0.180	0.001	− 0.354	− 0.259	0.005	− 0.046	− 0.083
O_2_					0.362	− 0.221	− 0.052	0.064	− 0.196	− 0.127	− 0.134	− 0.120	− 0.148
Na^+^						0.159	0.267	0.318	− 0.557	− 0.464	− 0.210	− 0.153	− 0.384
K^+^							− 0.341	− 0.231	− 0.247	− 0.459	− 0.045	− 0.092	0.180
PO_4_^3+^								0.6131	0.113	0.318	− 0.067	− 0.070	− 0.070
SO_4_^2−^									− 0.005	0.147	− 0.177	− 0.191	− 0.014
NO_3_^−^										0.706	0.450	0.451	**0.679**
NO_2_^−^											0.122	0.039	0.369
*E. coli*												**0.880**	**0.602**
ENT													**0.705**

**Table 8:** Spearman’s rank-order correlation of selected parameters [parameters include physicochemical parameters (pH, temperature (*T* °C), electrical conductivity (EC), dissolved oxygen (O_2_) and soluble ions (Na^+^, K^+^, PO_4_^3+^, SO_4_^2−^, NO_3_^−^ and NO_2_^−^) and faecal indicator bacteria (FIB): *Escherichia coli* (*E. coli*), *Enterococcus* (ENT), and Total Coliforms (TC). Significant coefficients (*P* < 0.05) are in bold] in water from shallow well analysed in dry season

	*T* (°C)	pH	EC	O_2_	Na^+^	K^+^	PO_4_^3+^	SO_4_^2−^	NO_3_^−^	NO_2_^−^	*E. coli*	ENT	TC
*T* (°C)		− 0.185	0.190	0.291	0.002	0.094	− 0.099	0.170	0.052	0.193	0.032	0.129	0.315
pH			0.149	− 0.358	− 0.120	0.120	0.014	− 0.407	− 0.097	− 0.027	0.338	0.433	0.233
EC				0.271	0.056	0.000	0.102	0.067	0.121	− 0.074	0.193	0.278	0.181
O_2_					0.287	0.1591	0.044	0.197	− 0.259	− 0.177	− 0.157	− 0.102	− 0.173
Na^+^						0.430	0.073	0.387	− 0.551	− 0.305	− 0.527	− 0.413	− 0.412
K^+^							− 0.023	− 0.002	− 0.524	− 0.168	− 0.112	0.110	− 0.089
PO_4_^3+^								− 0.008	0.009	− 0.048	0.269	0.140	0.065
SO_4_^2−^									0.299	0.157	− 0.192	− 0.233	0.028
NO_3_^−^										**0.692**	**0.593**	0.353	**0.635**
NO_2_^−^											0.437	0.219	**0.715**
*E. coli*												**0.902**	**0.812**
ENT													**0.785**
TC													

## Conclusion

The assessment of water physicochemical parameters and FIBs in the water sample means judging drinking water quality from any source. In this work, we have investigated the seasonal variations of physicochemical parameters and FIB levels in drinking water collected from wells and springs in Selembao and Kimbanseke, the city of Kinshasa in the DRC. To our best knowledge, this is the first study assessing wells and springs contamination by human faecal material in these municipalities. More than 80% of the domestic water supply in these municipalities comes from the investigated sources (wells and springs). The results revealed that except for NO_3_^−^ in 7 out of 21 examined water sources during the rainy season, the concentration of other investigated ions meets the WHO guidelines for drinking water quality during both dry and rainy seasons. As for bacteriological analysis, 60% (3 out of 5 managed/developed wells) are not contaminated by faecal material during dry and rainy seasons. Managed/developed wells represent less than 15% of the water supply sources in the Selembao and Kimbanseke municipalities. On the contrary, 100% of investigated unmanaged/undeveloped wells are heavily contaminated with faecal material in rainy and dry seasons. The findings of this study could indicate that unmanaged/undeveloped wells and springs studied are problematic and impact human health using these water sources for drinking purposes. The water samples from the investigated springs (except one spring during the dry season) present high faecal material contamination during both dry and rainy seasons. In fact, according to WHO drinking water regulations, water from many investigated sources is not appropriate for drinking or other domestic purposes. The contamination of water sources by microorganisms constitutes a significant public health risk because of their dangers to humans through consumption. These results corroborate our epidemiologic survey, which indicates the occurrence and persistence of waterborne diseases in the investigated municipalities. The absence of safely managed sanitation systems and services, poor governance, poverty, poor hygiene combined with the lack of water sources points protection are, therefore, at least responsible for the contamination of water sources by human faeces, and the occurrence of waterborne diseases in studied areas. Therefore, the results presented here provide baseline information and call for an urgent effort towards reducing the contamination of water sources by human faeces while maintaining epidemiological and laboratory surveillance on the quality of the authorities to inform and protect the population and practices. Nevertheless, further research on microbiological assessment of rivers, springers and wells is needed in different suburban communes of Kinshasa to fill a knowledge gap on water-related diseases. Moreover, the evaluation of other contaminants such as POPs, antibiotics, antibiotic-resistant bacteria and their resistance genes should also be investigated to evaluate water quality fully. Finally, we recommend to the local authority the monitoring programme of water quality, the population’s education programme, and the construction of appropriate wells for the people. In our view, some proactive measures such as piped drinking water supply, avoiding open defecation and establishing better toilet infrastructures, use of inexpensive bleach and access to sewers can mainly prevent surface water pollution. The approaches, methods, and scenarios used in this study can be applied in similar environments under tropical conditions to evaluate water quality regularly and prevent human health risks.
